# Quorum quenchers affect the virulence regulation of non-mucoid, mucoid and heavily mucoid biofilms co-cultured on cell lines

**DOI:** 10.1007/s00253-021-11638-8

**Published:** 2021-10-30

**Authors:** Rachith Kalgudi, Roya Tamimi, Godfrey Kyazze, Tajalli Keshavarz

**Affiliations:** School of Life Sciences, University of Westminster, 115 New Cavendish Street, London, W1W 6UW UK

**Keywords:** Quorum quenchers, Farnesol, Tyrosol, *Pseudomonas aeruginosa*, Acyl-homoserine lactones, Biofilms

## Abstract

Biofilm formation conferring pathogenicity is a survival strategy for *Pseudomonas aeruginosa*. *P. aeruginosa*’s virulence may differ due to differences in host-microbe interactions and the growth environment. The epithelial cell line within the respiratory system and the keratinocytes on the skin form the first physical barrier of defence. *P. aeruginosa* spp. biofilm formation and virulence factor secretion with and without quorum quenching (QQ) treatment was studied in co-culture using A549 and HaCaT cell lines; pyocyanin and rhamnolipid productions and elastolytic activity as virulence factors were quantified by independent assays. Biofilm formation was evaluated under dynamic conditions by quantifying total carbohydrates, alginate, proteins and eDNA. A sandwich ELISA was performed to study IL-8 secretion by the epithelial cells. The difference in gene expression of the quorum sensing (QS) and virulence factors between strains during individual and combination treatments was analysed by qPCR. Combination treatment by farnesol and tyrosol was more effective against *P. aeruginosa* biofilms when grown in co-cultures. The strain RBHi was found to be 3 to 4 times more virulent compared to PAO1 and NCTC 10,662, respectively, and combination treatment was more effective against RBHi strain when grown in co-culture with A549 cell line. The addition of quorum quenchers (QQs) individually and in combination reduced IL-8 secretion by A549 cells. Relative mRNA expression showed upregulation of the QS genes and virulence factors. Co-culture of *P. aeruginosa* and HaCaT cell line showed a general decrease in gene expression, especially in the case of *P. aeruginosa* RBHi when treated with farnesol and tyrosol combination.

**Key points**

• *Differentiating the interactions of biofilm formed by different phenotypes of P. aeruginosa, NCTC 10,662 (non-mucoid), PAO1 (semi mucoid) and RBHi (heavily mucoid).*

• *Biofilm formed by these P. aeruginosa strains on two commonly afflicted tissues represented by A549 (lung) and HaCaT (skin) cell lines.*

• *Anti-biofilm/anti-virulence roles of quorum quenchers, tyrosol and farnesol in co-cultures.*

**Supplementary Information:**

The online version contains supplementary material available at 10.1007/s00253-021-11638-8.

## Introduction

The Gram-negative bacterium, *Pseudomonas aeruginosa*, is a well-known opportunistic pathogen that causes genetically inherited human cystic fibrosis (CF). During chronic infections in CF airways, it is common for the bacterium to acquire selective phenotypes that aid its survival within the environment of the human lung in the presence of toxic, osmotic and oxidative stress induced by the host immune system (Camus et al. 2021). Literature has documented that in such a scenario, a primary selective phenotypic change is the production of alginate that envelops the bacteria (mucoid). This results in sticky mucus, most notably in the respiratory tract of CF patients making them susceptible to chronic lung infections and the high rate of morbidity and mortality associated with CF (Bhagirath et al. 2016).

The presence of non-mucoid, mucoid, and strongly mucoid strains of *P. aeruginosa* highlights the variety of genetic variation in this pathogenic bacterium (Workentine et al. 2013). *P. aeruginosa*’s ability to form biofilms, which is regulated by its ability to coordinate gene expression via the cell density-dependent mechanism of quorum sensing (QS), is the key virulence factor that causes chronic infections (Sousa and Pereira 2014). The ability of *P. aeruginosa* to form biofilms varies between strains and is reflected in the extracellular polymeric substances (EPS) composition and biofilm structure, which is affected by growth conditions and nutrient availability. In addition to external stimuli, the presence or absence of ancillary factors such as flagella, fimbriae and polysaccharide matrix production may influence the growth of biofilms and define their total biomass (Berne et al. 2015).

When considering the role of QS in pathogenic bacteria in the development of novel antibacterial therapies, the following QS-mediated features that make the pathogen resilient must be noted: colonisation, adhesion, acid tolerance, biofilm formation, motility, sporulation and other virulence factors vary by bacterial species (Rutherford and Bassler 2012; Obana et al. 2014; Abisado et al. 2018).

*P. aeruginosa* can adapt and survive in a number of environmental niches due to the existence of various regulatory proteins. The diversity of virulence factors (toxins) released by *P. aeruginosa* and its ability to form robust biofilms are important considerations (Moradali et al. 2017a, b). Biofilm formation along with secreted toxins aids *P. aeruginosa* in circumventing the immune response of the host (Moser et al. 2017). Each individual virulence factor plays a role in the pathogenesis of *P. aeruginosa*; for instance, elastase degrades the extracellular matrix proteins of epithelial cells and disrupts the blood vessels (Xu and Shi 2014), rhamnolipids promote invasion of *P. aeruginosa* by disrupting the host tissue (Alhazmi 2015), and pyocyanin inhibits cellular respiration (Hall et al. 2016).

The prevalence of certain virulence factors may change with the localisation of the infection in the host tissue by *P. aeruginosa* (Gellatly and Hancock 2013). As a result, *P. aeruginosa* pathogenesis is commonly linked to the expression and synthesis of a variety of cell-associated and extracellular virulence factors mediated by QS. In general, virulence of *P. aeruginosa* can be differentiated between non-mucoid, mucoid and strongly mucoid strains, with overproduction of alginate (Ryall et al. 2014). The primary differentiations of virulence between non-mucoid and mucoid strains include the loss of flagella and pili affecting motility and loss of pigment production. Subsequently, mucoid strains of *P. aeruginosa* are able to either proliferate and cause acute infections or propagate in the case of chronic infections (Valentini et al. 2018).

Internalisation of bacterial cells into the epithelium is advantageous to the host early in the infection as it triggers inflammation and an immune response (Lippmann et al. 2015). *P. aeruginosa* spp. are known to bind to epithelial cells and facilitate internalisation as a survival/defence mechanism against the host immune response (Lepanto et al. 2011). They adhere to epithelial cells with their flagellum and pili and enter the cells through receptor-mediated endocytosis (Cossart and Helenius 2014). According to studies, internalisation is critical in assisting the bacteria’s survival within the host tissue by avoiding the killing mechanisms.

Variation in mucoid isolates from chronic lower airway infections is often attributed to mutations in the alginate biosynthesis pathway (Sousa and Pereira 2014). To undergo a physiological transformation into pathogenic behaviour and cause infections in human hosts, an environmental strain of *P. aeruginosa* must produce alginate. Alginate provides a stable environment for the bacteria and aids in the creation of a mature biofilm’s three-dimensional structure (Roy et al. 2018). Alginate, which is predominantly found in mucoid strains of *P. aeruginosa*, is considered to be an anionic polymer, rich in mannuronic acid and glucuronic acid (Limoli et al. 2015). A very selective phenotype of alginate overproduction is observed in highly mucoid CF isolates (Limoli et al. 2017). Folkesson et al. (2012) found that the existence of sub-lethal levels of hydrogen peroxide, a product of polymorphonuclear neutrophils, is linked to the overproduction of alginate in CF isolates. In a related research, Mathee et al. (1999) discovered that non-mucoid strains produce/overproduce alginate in the presence of hydrogen peroxide.

The development of IL-8 by epithelial cells is a key indicator of the host’s reaction, and it is usually linked to both acute and chronic infections (Shahzad et al. 2010). IL-8 development from host tissue is stimulated by a variety of bacterial secreted factors (Kobayashi et al. 2018). In the presence of biofilm-related infections, the presence of flagellin and lipopolysaccharides and the continuous synthesis and release of N-acyl-homoserine lactones (AHL) molecules, have been identified as the key causes of IL-8 secretion by mammalian cells (Li and Tian 2012a, b).

*P. aeruginosa* and *Candida albicans* are known to coexist in a clinical setting and are commonly found it the respiratory tract in CF airways (Haiko et al. 2019). The ability to form biofilm by *C. albicans* is dictated by its propensity to produce QS molecules such as farnesol and tyrosol (Monteiro et al. 2017).

Farnesol and tyrosol function primarily as fungal QS molecules, whereby they aid in regulating the gene expression and help coordinate collective behaviour of fungi to thrive in their niche (Rodrigues and Černáková, 2020). As a fungal QS molecule, the primary function of farnesol is to inhibit the yeast hyphae formation in a concentration-dependant manner, whereas tyrosol stimulates hyphae and germ tube formation in a concentration-dependant manner. Collectively, farnesol and tyrosol function in tandem as required in the life cycle of the fungi to aid in fungi adhesion, proliferation, filamentation and dispersal (Zawrotniak et al. 2019 and Rapala-Kozik et al. 2019).

Compared to abiotic surfaces, the use of cell lines allows for the creation of experimental designs that can address basic mechanical and biochemical questions to help in the understanding of what happens in a regulated in vitro setting. This could help researchers figure out what QQ does and how it affects QS-mediated gene expression, which facilitates bacterial contact and biofilm formation. There is little information in literature investigating the effect of QQ on biofilm formation by different phenotypes of *P. aeruginosa* on a biotic surface (Chien Yi 2018; Ruiyi et al. 2019; Thi et al. 2020). Our study made use of the A549, human lung carcinoma cells (Kaplan et al. 2017) and HaCaT, immortalised human keratinocyte cells (Seo et al. 2012) to investigate for the first time the variation in biofilm formation and virulence factor secretion between non-mucoid, mucoid and a heavily mucoid cystic fibrosis (CF) isolate of *P. aeruginosa* in the presence of QQs, farnesol and tyrosol.

In vitro studies have shown QS-mediated changes in *P. aeruginosa* in relation to regulation of gene expression, specifically during biofilm formation. Production of virulence can be differentiated, as mentioned above, between non-mucoid and mucoid strains of *P. aeruginosa*. Furthermore, downregulation of certain virulence factors has been reported when comparing non-mucoid and mucoid strains of *P. aeruginosa* (Lee et al. 2005 and Moradali et al. 2017a, b).

However, most of the in vitro studies conducted so far do not take into consideration a scenario whereby QS molecules of other species of microorganisms coexist alongside *P. aeruginosa* in CF airways and their effect on biofilm formation and QS of *P. aeruginosa*. Additionally, the QQ effect of fungal QS molecules and their inhibitory effect against *P. aeruginosa* in the presence of mammalian cell lines are not reported.

Analysing *P. aeruginosa* biofilm formation and corresponding bacterial communication inhibition by (QQs) on mammalian cell lines will provide a better understanding of the phenomenon of QQ since it will enable researchers to test the efficacy of QQ in an environment that mimics a natural (biotic) surface, such as an epithelial cell line, for bacterial adherence and biofilm formation.

This study focuses on farnesol and tyrosol and assesses their ability to inhibit, as fungal QS molecules, the QS process in *P. aeruginosa*, and as QQs when grown on mammalian cell lines.

## Material and methods

### Bacterial strains and preparation of bacterial inoculum

*P. aeruginosa* NCTC 10,662 (non-mucoid), *P. aeruginosa* PAO1 (semi mucoid) and *P. aeruginosa* RBHi (heavily mucoid) were selected as model phenotypes for this study.

*P. aeruginosa* PAO1 and *P. aeruginosa* NCTC 10,662 were obtained from the University of Westminster, London, culture collection. *P. aeruginosa* CF isolate was kindly donated by the culture collection facility at Royal Brompton Hospital, London, UK (referred to as RBHi in this study).

*P. aeruginosa* strains were subcultured into Lysogeny broth (LB broth) from the slants and incubated aerobically overnight (16–18 h) at 37 °C at 180 rpm. The absorbance (OD_600_) of the overnight growth of *P. aeruginosa* sp. was readjusted in sterile LB broth to obtain an equivalence absorption according to 0.5 McFarland standards (~ 1.5 × 10^8^ cells) and used for further biofilm growth. A flow cell (Transmission Flow cell, FC 281, BioSurface Technologies Corporation (BST), Montana, USA) provided a closed system where bacterial attachment occurred and biofilm structure and hence EPS produced.

### Cell culture

A549 cells were obtained from the American Type Culture Collection (ATCC CCL-185). HaCaT cells were obtained as a kind donation from Division of Surgery and Interventional Science, UCL, Royal free Hospital, London, UK.

### A549 and HaCaT culture

A549 and HaCaT cells were seeded into either 96-well plate at 1 × 10^4^ cells per well or 6-well plate (Nunc) at 5 × 10^5^ cells per well, unless otherwise indicated. The cells were allowed to adhere overnight in high glucose DMEM (phenol red) supplemented with 10% FBS and 1 × pen strep. The plates were incubated at 37 °C at 5% CO_2_ for a period of 4 days until they reached ~ 80% confluency. The wells were then rinsed with 1 × PBS (phosphate-buffered saline) to wash away dead cells and phenol red prior co-culture experiments and treatments.

### Co-culture of P. aeruginosa sp. with A549 and HaCaT cell lines

Protocol for the co-culture biofilm model was adopted from Moreau-Marquis et al. (2010) with a few modifications. For the purpose of the assays, A549 and HaCaT cells were grown to confluence in 6-well tissue culture plates prior to bacterial inoculation. The three strains of *P. aeruginosa* were grown overnight in LB medium at 37 °C. The overnight bacterial growth was centrifuged at 5000 rpm to pellet the cells, and the supernatant containing the spent medium was discarded. The pellet containing the bacterial cells was resuspended in 1 × PBS. Based on bacterial cell count and seeded A549 and HaCaT cells, multiplicity of infection (MOI) was approximately adjusted to 20:1, and the co-culture was incubated for 1 h with and without treatment involving farnesol (E, E–farnesol) and tyrosol (2,4-hydroxyphenyl-ethanol) (Cambridge biosciences, Cambridge, UK) and MIC_50_ combination of the two as detailed in the relevant sections below. After the incubation period, the A549 and HaCaT cells were rinsed, and adhered bacteria were quantified by spot plate analysis based on work done by Thomas et al (2015). The percentage adherence was calculated based on the ratio of adhered bacteria to the number of bacteria in the inoculum. Internalisation assay involving the three strains followed a similar protocol as the adherence assay. Gentamicin at 200 mg/mL was used to eliminate the bacteria in suspension, and the A549 and HaCaT cells were lysed open.

### Cell viability assay (MTT assay)

Cell viability assay was performed based on the manufacturer’s instructions. For the purpose of the assay, the cells were treated after reaching confluency (~ 3 to 4 day) for a period of 4 h prior to performing the MTT assay. The growth medium from the experimental set-up from the wells (96-well plate) was removed and replaced with 100 μL of fresh medium. To the wells, 10 μL of the 12-mM MTT (Vybrant MTT Cell Viability Assay, Thermo Fisher Scientific) stock solution was added. A negative control was included by adding 10 μL of MTT stock solution to blank wells containing medium alone. After labelling the cells with MTT, 25 μL of the medium was removed and replaced with 50 μL of DMSO with thorough mixing by pipetting. The 96-well plates were then incubated at 37 °C and 5% CO_2_ for a period of 3 h. The samples were mixed again prior to reading the plate at 540 nm.

The mean of three independent experiments (*n* = 8) was used to calculate the cell viability. Percentage viability was calculated as the ratio between untreated and treated cells (and in co-culture). The wells without the cells and without treatment were used as the negative controls and the values subtracted from the negative control wells and treatment wells prior to analysis.

### Adherence and internalisation of P. aeruginosa sp. cells to A549 and HaCaT cells in co-culture

The adherence and internalisation of *P. aeruginosa* cells to A549 and HaCaT cells were compared as described previously by Carterson et al. (2005). To identify the adherence of bacterial cells, co-culture was carried out as described previously, with and without treatment**.** Following the required incubation time, the plates containing the A549 and HaCaT cells were washed with 1 × PBS, and 200 mL of PBS-T was introduced in the wells and incubated for 30 min. Following incubation, the medium was then removed and spot plated for CFU enumeration. Percentage adherence was calculated based on CFU obtained from sessile cell count against the initial CFU of the inoculum used.

Gentamicin exclusion assay was adopted to calculate the internalisation of the bacteria. Following incubation, the external bacteria were killed using 200 mg/mL of gentamicin for 2 h. Controls of A549 and HaCaT cell free wells were used as controls to show complete killing of external bacteria. After 2 h, gentamicin was washed away, three times, by rinsing with 1 × PBS. The A549 and HaCaT cells were then lysed by adding 1% Triton X-100 in PBS for 1 h, and the plates were incubated at 37 °C. Following incubation, the medium from the plates were spot plated to obtain CFU counts. Percentage internalisation was calculated based on the CFU obtained after gentamicin treatment against the initial CFU count of the inoculum.

### Quantification of P. aeruginosa sp. biofilm by CV staining method

Numerous in vitro models have been suggested to investigate and quantify biofilm formation (Lebeaux et al. 2013). In a 96-well microtiter tray, 200 μL of adjusted inoculum of *P. aeruginosa* sp. was added to all test wells and 200 μL of the appropriate growth medium to control wells. After incubation for a period of 4 h, the wells were gently washed with PBS to remove unattached planktonic cells, and the appropriate amount of medium was re-added for biofilm growth. Under static conditions, the 96-well microtiter plates were incubated at 37 °C for a period of ~ 18 h. After incubation for biofilm growth, biofilm was fixed using heat fixation 60 °C for 60 min. Crystal violet (CV) (220 μL volume, 0.1% w/v) was added to each well and allowed to stain for 10 min at room temperature. The optical density was determined at 570 nm using VersaMax Tunable Microplate Reader (VWR, Leicestershire, UK).

In order to prevent overestimation of the quantity of biofilm, the absorbance value obtained with CV staining of the control A549 cells was subtracted from the experimental values prior to analysis. Individual negative control of A549 cells was included in each of the experimental set-up undergoing the same treatment without the inclusion of bacterial cells. The same was performed for all biochemical analysis of biofilm components and virulence production. All experiments were performed in triplicates.

### Analysis of EPS composition of P. aeruginosa sp. biofilms

Based on work conducted by Liu and Fang (2002), the protocol for formaldehyde-NaOH method of EPS extraction was adopted for this study with a few modifications.

#### Quantification of total carbohydrates

The method described by Dubois et al. (1956) was adopted to quantify the total sugars present in the EPS extracted from the biofilm formed by *P. aeruginosa*. Reagents used for the total carbohydrate assay were phenol (80%w/v) and sulphuric acid (95.5% reagent grade). Absorbance was recorded at 490 nm. A standard curve was prepared using glucose. A modified version of the phenol–sulphuric acid assay as described by Masuko et al. (2005) was adopted for this study due to the convenience of performing the protocol and also due to the numerous samples that needed to be analysed. The high-throughput nature of the assay and use of phenol and sulphuric in limited quantities were desirable.

#### Quantification of alginate

Carbazole reaction-mediated quantification of uronic acid content was performed using D-glucuronic acid lactone as standard. A version of the assay based on Bitter and Muir (1962) and modified by Cesaretti et al. (2003) was used.

#### Quantification of total proteins

A microtiter plate-based Bradford assay was adopted for this study, as modified by Ernst and Zor (2010). The modifications were based on the Bradford assay that makes use of the Coomassie brilliant blue stain. All experiments were performed in triplicates, and the experiments were repeated three times for reproducibility.

#### Quantification of eDNA

eDNA content of the biofilms formed by *P. aeruginosa* sp. was quantified by a fluorometric assay using a dsDNA Qubit Broad-Range assay kit (Thermo Fisher Scientific, Dartford, UK).

### Analysis of virulence factors

#### Quantification of elastolytic activity

The elastolytic activity of *P. aeruginosa* sp. was determined using elastin Congo red (ECR) assay as described by Pearson et al. (1997) with modifications. Aliquots (100 mL) of biofilm extracts were added to tubes containing 20 mg ECR in 900 mL of ECR buffer (100 mM Tris, 1 mM CaCl_2_, pH 7.5). The mixture was then incubated at 37 °C for a period of 3 h and incubated on ice after 0.1 mL of 0.1 M EDTA and was added. Insoluble ECR was removed by centrifugation, and the absorbance of the supernatant was measured at 495 nm.

#### Quantification of pyocyanin

Pyocyanin quantification was performed based on the method by Essar et al. 1990 with modifications. Quantification was based on absorbance of pyocyanin at 520 nm under acidic conditions after phase separation using chloroform. Biofilms were resuspended in 1 × PBS, and 500 mL of the resuspension was extracted with 3 mL of chloroform and re-extracted into 2 mL of 0.2 M HCl to provide a pink to red solution. Sample volumes (200 mL) were then transferred to a microtiter plate, and the absorbance was measured at 520 nm. The concentration of pyocyanin was expressed as mg/mL upon multiplying the absorbance at 520 nm with the molar extinction coefficient of 17.072 (Moayedi et al. 2017).

#### Quantification of pyoverdine

Spectrofluorometric quantification was performed by measuring the resuspended biofilm filtrate (0.22-mm filter) at 405 nm.

#### Quantification of rhamnolipid

Quantification of rhamnolipid was carried out by applying the orcinol reaction as described by Laabei et al. (2014) with modifications. The supernatant was extracted 3 times with 1 mL diethyl ether prior to complete evaporation under vacuum. Upon completion, 0.5 mL of distilled H_2_O was added to each of the sample tubes. For the assay, 100 mL of samples was taken after resuspension in dH_2_O. To the samples, 900 mL of 0.19 orcinol (diluted in 53% H_2_SO_4_) was added and incubated at 80 °C for 30 min. After incubation, the samples were cooled to room temperature, and the absorbance was measured at 421 nm and the concentration was measured based on the standard curve prepared using rhamnose.

### IL-8 secretion analysis in P. aeruginosa sp. Biofilm co-cultured with A549 and HaCaT cells

IL-8 secretion by A549 and HaCaT cells following biofilm formation by *P. aeruginosa* sp., with and without treatment, was performed. Following co-culture and treatment with farnesol and tyrosol in 6-well plates, the supernatant of the co-culture was removed and stored at − 80 °C until further use. A sandwich ELISA was performed using the Human IL-8 ELISA kit (Invitrogen, Paisley, UK). Protocol followed was based on the manufacturer’s instruction.

### qPCR analysis of QS network and virulence factors in P. aeruginosa co-cultured with A549 and HaCaT cells

RNA extraction from bacterial cells was performed using the TRIzol RNA isolation protocol described by Chomczynski and Sacchi in 1987. cDNA preparation was then done by using the QuantiTect Reverse Transcription kit (Qiagen Ltd, Manchester, UK).

Samples prepared by using diluted forward and reverse primer (Tables 1 and 2) mix (1/10 dilution) run in duplicate and the conditions were set as follows: Initial denaturation was done at 95 °C for 2 min. Subsequently, the amplification program involved 40 cycles of denaturation at 95 °C for 15 s, primer annealing at 55 °C for 15 s and extension at 72 °C for 30 s. A final extension was performed at 72 °C for 2 min followed by cooling at 4 °C. A dissociation step at 55 °C was used to generate a melting curve with a 1 °C increase every 5 s until 95 °C to obtain verification of amplified product. Reference genes were as reported in Table 3. SYBR Green qPCR was performed using Rotor Gene Q (Qiagen Ltd, Manchester, UK).

**Table 1:** Primers for QS genes in *P. aeruginosa*

Gene	Primer	Nucleotide sequence	Reference
*lasI*	Forward	5′ CGTGCTCAAGTGTTCAAGG 3′	Jack et al. (2018); Zhu et al. (2004)
	Reverse	5′ TACAGTCGGAAAAGCCCAG 3′	
*lasR*	Forward	5′AAGTGGAAAATTGGAGTGGAG 3′	
	Reverse	5′ GTAGTTGCCGACGACGATGAAG 3′	
*rhlI*	Forward	5′ TTCATCCTCCTTTAGTCTTCCC 3′	
	Reverse	5′ TTCCAGCGATTCAGAGAGC 3′	
*rhlR*	Forward	5′ TGCATTTTATCGATCAGGGC 3′	
	Reverse	5′ CACTTCCTTTTCCAGGACG 3′

**Table 2:** Primers for virulence factors of *P. aeruginosa*

Gene	Primer	Nucleotide sequence	Reference
*toxA*	Forward	5′ GGAGCGCAACTATCCCACT 3′	Sabharwal et al. (2014); Aghamollaei et al. (2015)
	Reverse	5′ TGGTAGCCGACGAACACATA 3′	
*aprA*	Forward	5′ GTCGACCAGGCGGCGGAGCAGATA 3′	
	Reverse	5′ GCCGAGGCCGCCGTAGAGGATGTC 3′	
*rhlAB*	Forward	5′ TCATGGAATTGTCACAACCGC 3′	
	Reverse	5′ ATACGGCAAAATCATGGCAAC 3′	
*lasB*	Forward	5′ TTCTACCCGAAGGACTGATAC 3′	
	Reverse	5′ AACACCCATGATCGCAAC 3′

**Table 3:** Reference genes for q-PCR

Gene	Primer	Nucleotide sequence	Reference
*rpsL*	Forward	5′ CCTCGTACATCGGTGGTGAAG 3′	Pourakbari et al. (2016); Jack et al. (2018)
	Reverse	5′ CCCTGCTTACGGTCTTTGACAC 3′	
*AmpC*	Forward	5′GGTGCAGAAGGACCAGGCACAGAT 3′	
	Reverse	5′CGATGCTCGGGTTGGAATAGAGGC 3′	

### Statistical analysis

All experiments were performed in triplicates. All data for assays performed in this study were statistically analysed using GraphPad Prism to determine *p* values and establish correlation between data sets. All graphs were plotted using GraphPad Prism. *p* < 0.05 was considered significant.

## Results

### *P. aeruginosa* sp. adherence pattern to A549 and HaCaT cell lines with and without QQ mediated by farnesol and tyrosol

Cell viability assay involving MTT reagent was performed on A549 and HaCaT cells to see if farnesol and tyrosol displayed any antagonistic activity at the concentrations used. As A549 and HaCaT cells do not tolerate the MIC_50_ of farnesol and tyrosol used for bacterial culture (Fig. S1a), a significantly lower concentration was used (Fig. S1b). MTT assay was performed on sub-MIC_50_ of farnesol (2.5 µM) and tyrosol (1.5 µM) as single treatments and on the sub-MIC_50_ of each in combination, farnesol (1.25 µM) and tyrosol (0.75 µM), respectively; the assay did not show decrease in cell viability of both A549 and HaCaT cell lines (Fig. S1c).

However, MTT assay did reveal a significant decrease in cell viability of both the cell lines when grown in co-culture with *P. aeruginosa* sp. (Fig. S2). Overall viability of HaCaT cells was found to be lower when compared to A549. In order to understand the relevance of these findings, assays were performed to identify bacterial adherence patterns to the mammalian cell lines as well as the ability of the bacterial cells to internalise into the epithelial cells.

Compared to the control (untreated samples), cell viability for both cell lines in co-cultures decreased significantly as expected (Fig. S2). A greater reduction in overall cell viability was seen with HaCaT cells compared to A549 cells. Cell viability for farnesol and tyrosol and in combination showed a small decrease in viability but was not found to be significant (*p* = 0.532). Bacterial adherence to A549 and HaCaT cell lines was determined by spot plating and by determining the ratio between the number of bacterial cells in the inoculum to the number of bacteria adhered to the mammalian cells. Neither of the strains showed a stronger affinity to adhere to the mammalian cells when compared to the controls. The main difference, a higher percentage of adhesion to HaCaT cells, was observed when compared to A549 cells (*p* = 0.002) as shown in Figure S3; it was promising to see the antagonistic effect of farnesol and tyrosol on reducing adhesion. Greater amount of adherence from the CF isolate *P. aeruginosa* RBHi was expected. However, it was not the case and difference in adhesion, based on a two-way ANOVA that did not show significance (*p* = 0.325).

The adherence pattern was found to be similar between the three *P. aeruginosa* strains and the two mammalian cell lines used in this study, as seen in Figure S3. Therefore, comparison of bacterial internalisation between the two cell lines can be beneficial for the infected host as it would most likely trigger a quick immune response to combat the infection during the early stages rather than later stages with an established biofilm-based infection (Eisenreich et al. 2013).

Results indicated a higher percentage of internalisation compared to adherence of *P. aeruginosa* sp. onto A549 compared to the HaCaT cell line (Figs. S3 and S4). Dunnett’s multiple comparison test (two-way ANOVA) showed that the non-mucoid strain *P. aeruginosa* NCTC 10,662 exhibits a lower percentage of internalisation (*p* = 0.001) compared to both PAO1 and the CF isolate RBHi in co-culture with A549 cell line as shown in Figure S4a. However, no significant decrease or increase in internalisation was observed (*p* = 0.7538) between mucoid and non-mucoid strains against HaCaT (Fig. S4b).

Although the internalisation process was inhibited in NCTC 10,662 by all the treatments, only the combination treatment of farnesol and tyrosol was responsible for a significant reduction in internalisation in A549 cells. Upon treatment with farnesol, tyrosol and a sub-MIC_**50**_ combination of the two, a decrease in attachment was observed amongst all the strains. The highest decrease was seen with the non-mucoid strain NCTC 10,662 on the A549 cell line with the combination treatment and individual treatment alike (*p* = 0.0001). This indicates that the propensity for *P. aeruginosa* to internalise when grown on A549 cell line is more prevalent than adherence during initial colonisation when still in planktonic/sessile stage of existence.

This indicates that the potential virulence mechanism of internalisation in A549 cells is more prevalent during initial colonisation compared to adherence.

*P. aeruginosa* PAO1 on the HaCaT cells showed a significant decrease in attachment with individual treatments of farnesol and tyrosol (*p* = 0.0044) and with combination treatment (*p* = 0.0001) (Fig. S3b). However, only the combination treatment of farnesol and tyrosol was able to elicit a significant decrease in bacterial cell internalisation with the CF isolate and A549 cell line (Fig. S4a). In comparison, a significant decrease in bacterial cell internalisation was observed with tyrosol and farnesol, when administered individually and when administered in combination specifically with the non-mucoid *P. aeruginosa* NCTC 10,662 (*p* = 0.021 and 0.0047, respectively) (Fig. S4a).

### Inhibition of P. aeruginosa sp. biofilm formation and virulence factor production on A549 and HaCaT cell lines by farnesol and tyrosol, individually and in combination

Time-point inoculation experiments were performed to quantify biofilm formation/inhibition and virulence factor production by *P. aeruginosa* sp. The co-culture medium contained the MIC50 of farnesol and tyrosol relevant to A549 and HaCaT cells.

CV assay was performed to quantify biofilm formation of *P. aeruginosa* sp. in co-culture with A549 and HaCaT cell lines (Supplemental Figs. S5 and S6). Referring to supplemental Figure S5, farnesol and tyrosol, individually and in combination, have a reduced effect on total biofilm formation in co-culture when compared to their inhibitory effect on just bacterial biofilm formation. The reduced effect can be attributed to the MIC_50_ of farnesol and tyrosol used for co-culture experiments, which are less than those in bacterial cultures. Between the two cell lines, biofilms grown on A549 generally showed a greater EPS content.

Though the addition of farnesol, tyrosol and combination reduced overall biofilm formation, a significant reduction of biofilm formation was observed with the addition of combination of farnesol and tyrosol (sub-MIC) for the non-mucoid, mucoid and heavily mucoid strains (*p* = 0.0001, 0.0036 and 0.0001, respectively); *P. aeruginosa* RBHi showed ~ 26% reduction in total biofilm formation, which was the highest amongst all the three strains grown in co-culture. The total carbohydrate content of the biofilms in co-culture did not show a significant decrease with the addition of farnesol for all the three strains. A reduction in alginate content of the EPS produced by *P. aeruginosa* RBHi was seen (*p* = 0.0012). Conversely, tyrosol showed a reduction in total carbohydrate content with the RBHi strain without any significant reduction in alginate content, when compared to the treatment with farnesol. The combination treatment saw a significant (*p* = 0.001) reduction in total carbohydrate content to be significant. However, alginate production was greatly reduced in PAO1 and RBHi (*p* = 0.0002 and 0.0001, respectively). eDNA and protein content on *P. aeruginosa* sp. biofilm remained unaltered with farnesol; however, a reduction in protein content was seen with the addition of tyrosol to the co-culture with PAO1 and RBHi (*p* = 0.0025 and 0.012, respectively). Combination treatment showed a reduction in eDNA and protein content amongst all the strains of *P. aeruginosa*; however, only the reduction in protein content with the combination treatment was found to be significant by a two-way ANOVA (*p* = 0.0167). In comparison, co-culture with HaCaT cells showed significantly lower quantity of total biofilm formation via the CV assay across all *P. aeruginosa* strains and treatments (Supplemental Fig. S6) compared to co-culture with A549 (two-way ANOVA, *p* = 0.0001).

According to supplemental Figure S6, following the trend seen in A549 co-culture, the treatments with farnesol, tyrosol and combination were found to be more effective in inhibiting biofilm formation in co-culture with HaCaT cell line, compared to their respective controls. With the exception of NCTC 10,662, the eDNA and protein content of the biofilm produced by the mucoid strain on the HaCaT cell line against all three treatments remained identical to the control. A similar trend was observed in the total carbohydrate content of the biofilm. Tyrosol and combination were found to be more effective in reducing total carbohydrate content of the biofilm (*p* = 0.025 and 0.0001, respectively).

**Fig.1:**
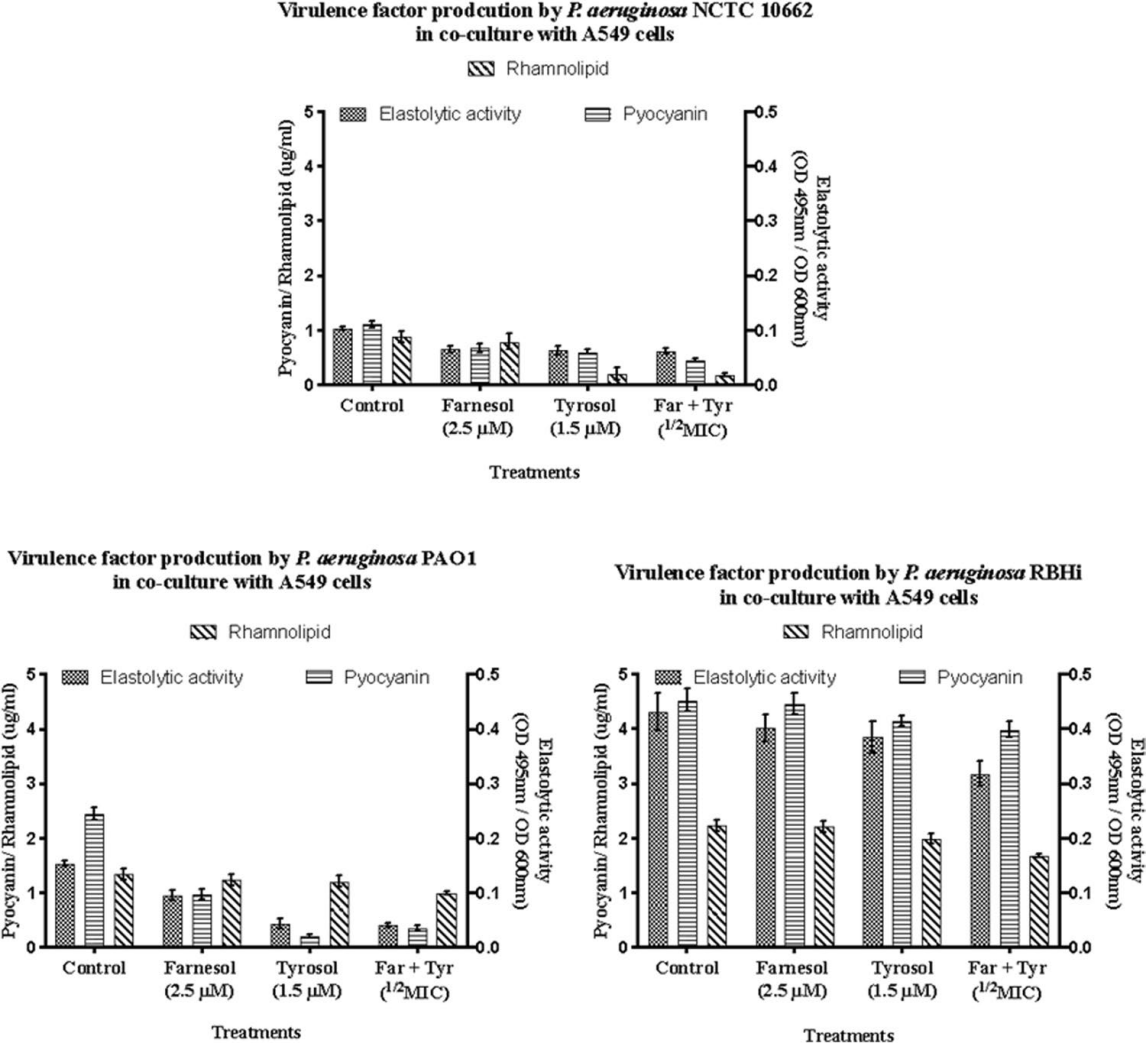
Virulence factor production by *P. aeruginosa* strains in co-culture with A549 cell line (*n* = 5). Error bars represent standard deviation

Virulence factors such as rhamnolipid, elastolytic activity and pyocyanin production were quantified by independent assays. Compared to the untreated control, a reduction of total virulence was seen with all three treatments involving farnesol, tyrosol and combination (Fig. 1). Reduction of elastolytic activity and pyocyanin production using farnesol was significant (*p* = 0.001 and 0.0001 respectively) in *P. aeruginosa* NCTC 10,662. However, the decrease in rhamnolipid production was not significant. Use of tyrosol showed a tremendous decrease (*p* = 0.0001) in rhamnolipid production compared to the treatment with farnesol, while no significant decrease was seen in elastolytic activity and pyocyanin production.

Comparatively in PAO1, all the treatments showed a reduction in elastolytic activity and pyocyanin production (*p* = 0.0001), while only the combination treatment showed a significant decrease in rhamnolipid production (*p* = 0.0024), and reduction in rhamnolipid production by individual treatments was not significant (*p* = 0.832). Comparing the individual treatments, tyrosol was more effective in reducing elastolytic activity and pyocyanin production compared to farnesol (*p* = 0.0001).

*P. aeruginosa* RBHi was found to be 3 to 4 times more virulent compared to PAO1 and NCTC 10,662, respectively, and combination treatment showed a significant decrease (*p* = 0.0001) in rhamnolipid production and elastolytic activity compared to the individual treatments and the control. Though pyocyanin production was reduced with the combination treatment compared to the control, it was not significant compared to the individual treatments (*p* = 0.735). Overall, the combination treatment involving farnesol and tyrosol was more effective against the highly virulent RBHi strain when grown in co-culture with A549 cell line.

**Fig.2:**
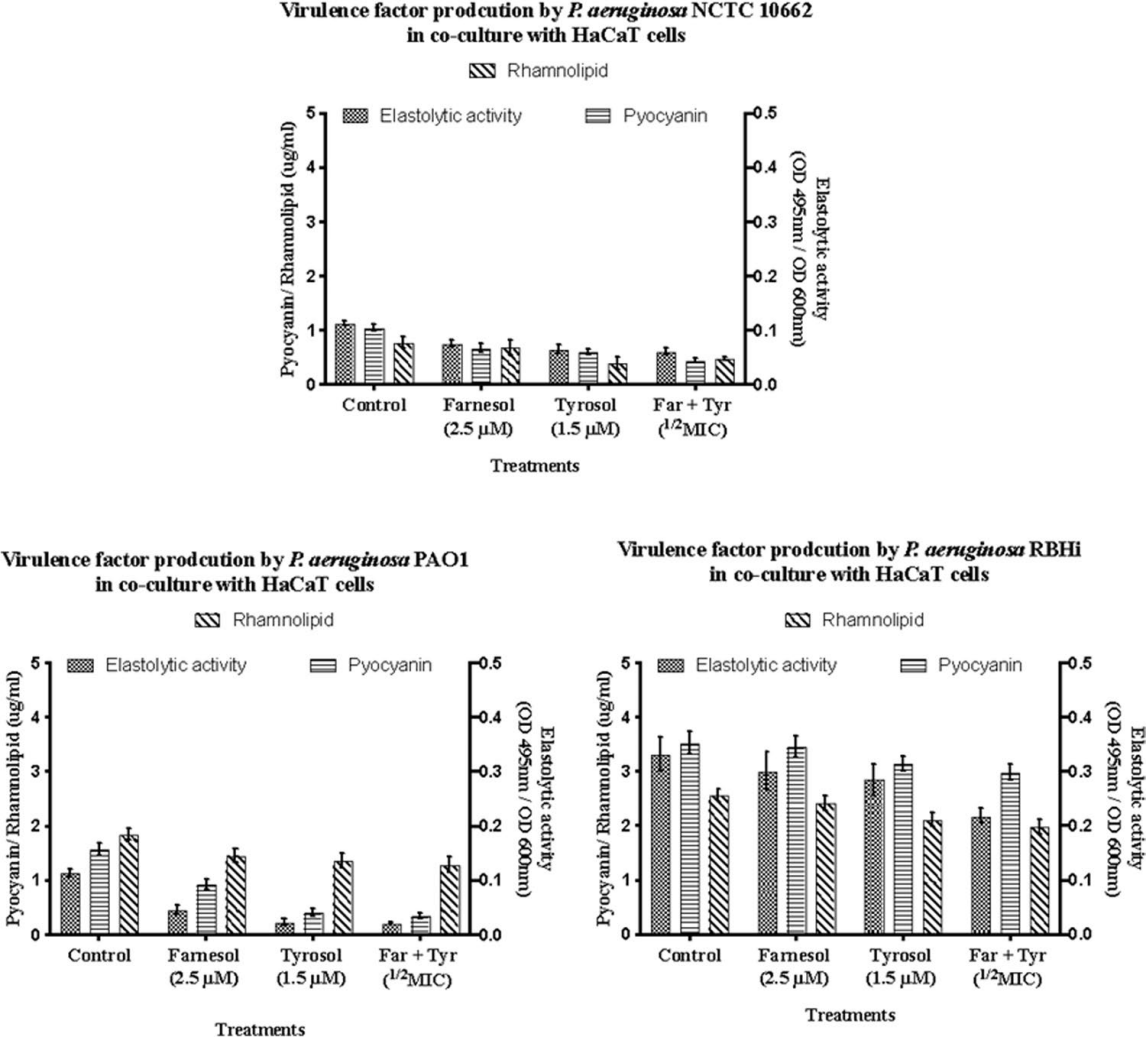
Virulence factor production by *P. aeruginosa* strains in co-culture with HaCaT cell line (*n* = 5). Error bars represent standard deviation

Quantification and analysis of virulence factors produced by *P. aeruginosa* sp. biofilms grown on HaCaT cell line showed a different profile compared to virulence produced upon co-culture with A549 cell line. In the non-mucoid strain, *P. aeruginosa* NCTC 10,662, combination treatment with farnesol and tyrosol was found to be the most effective at reducing the production of virulence; however, all treatments reduced the production of virulence significantly (*p* = 0.0032, Bonferroni post hoc test). In *P. aeruginosa* PAO1, elastolytic activity and pyocyanin production were reduced significantly with all treatments (*p* = 0.0001); however, rhamnolipid production remained at elevated levels even though it did reduce in comparison with the control (*p* = 0.002). Overall, treatment with tyrosol and in combination was found to be more effective (Fig. 2).

The CF isolate *P. aeruginosa* RBHi showed higher levels of virulence when compared to PAO1. These two mucoid strains showed a similar trend in decrease of virulence when treated with QQs. Reduction of rhamnolipid, pyocyanin and elastolytic activity was found to be significant (*p* = 0.0001) when compared to their respective untreated internal control. However, compared to NCTC 10,662, their virulence was elevated.

**Fig.3:**
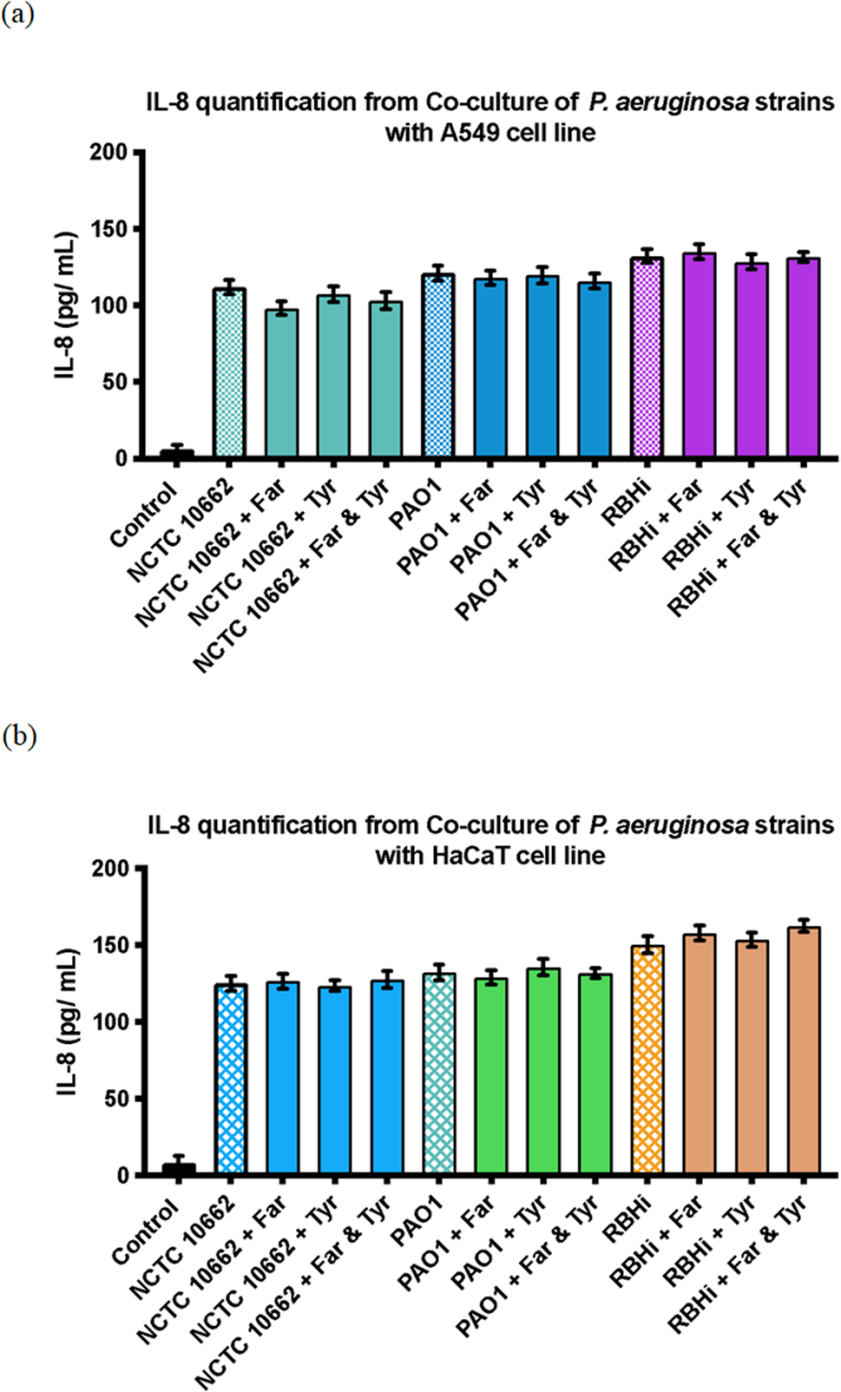
*P. aeruginosa* sp. biofilm formation stimulates significantly increased levels of IL-8 release, compared to the control, in **a** A549 cells when exposed to biofilm formation and treatment with farnesol and tyrosol and in **b** HaCaT cells when exposed to biofilm formation and treatment with farnesol and tyrosol. Secreted IL-8 was measured by ELISA (*n* = 3). Error bars represent standard deviation

A549 and HaCaT cells were grown and exposed to biofilms formed by *P. aeruginosa* NCTC 10,662, PAO1 and RBHi. Time-point inoculation studies also included farnesol and tyrosol as individual treatments as well as combination treatment to quantify IL-8 secretion by preventing biofilm formation. From ELISA, it was found that there was an increase in IL-8 secretion by A549 cells, which increased in the presence of biofilm formed by *P. aeruginosa* (Fig. 3a). The exogenous addition of farnesol and tyrosol and combination to combat biofilm formation did show a reduced IL-8 secretion. In the case of NCTC 10,662, the reduction in IL-8 production when treated with farnesol was found to be significant (*p* = 0.044), while the others were not found to be significant. In the case of HaCaT cells, reduction of IL-8 was not observed under any set of treatments with all the three *P. aeruginosa* strains (Fig. 3b). However, the basal level of IL-8 secretion for both the cell lines (A549 and HaCaT) was very low in the absence of stimulation along with low levels of apoptosis. This is generally expected of cell lines and was therefore found suitable as mammalian cell models for this study.

Production of IL-8 by HaCaT and A549 cells in the presence of *P. aeruginosa* and also when treated with farnesol and tyrosol individually and in combination was not significant (Fig. 3).

**Fig.4:**
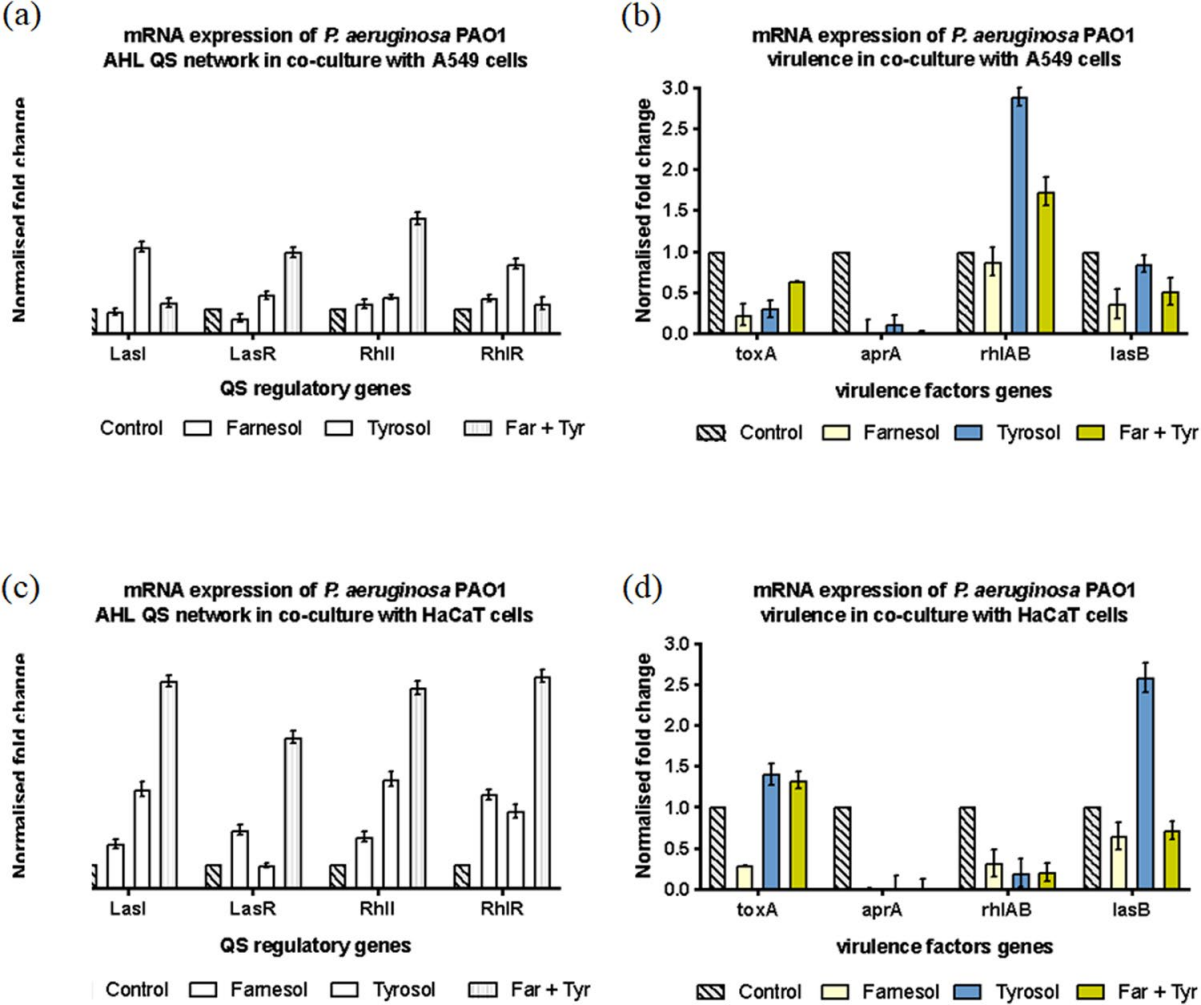
mRNA expression of AHL-mediated QS circuit and virulence factors *in P. aeruginosa* PAO 1 sessile cells grown with QQ. The fold change of mRNA for QS and virulence genes was determined for *P. aeruginosa* PAO1 sessile cells extracted from biofilm treated with farnesol and tyrosol after ~ 16 h growth. Results are expressed as the mean fold change (control standardised to 1.0) with error bars representing SEM (*n* = 9). Error bars represent standard deviation

Following farnesol, tyrosol and combination treatment of *P. aeruginosa* PAO1 biofilm in co-culture with A549 and HaCaT cell lines, the LasI/R and RhlI/R genes were significantly upregulated compared to the control (Fig. 4a, c). Comparing the two cell lines, co-culture with HaCaT cell line showed a significantly higher LasI/R and RhlI/R upregulation compared to co-culture on A549 cell line. While combination of farnesol + tyrosol combination induced a significant upregulation of LasR and RhlI on A549 cells, individual treatment with tyrosol was responsible for upregulation of LasI and RhlR (Fig. 4a). Co-culture of PAO1 with A549 cell line treated with farnesol showed a downregulation of *toxA*, *aprA*, *rhlAB* and *lasB* virulence. However, tyrosol and combination treatment showed a tremendous upregulation of *rhlAB* in PAO1 co-culture with A549 cell line (Fig. 4b), while all others were downregulated with tyrosol and combination treatment. Individual treatment with farnesol downregulated LasR in co-culture with A549 cell line. Combination treatment with farnesol + tyrosol showed a consistent and significant upregulation of LasI/R and RhlI/R of PAO1 in co-culture with HaCaT cell line (Fig. 4c). Individual treatment with farnesol and tyrosol showed significant upregulation of LasI/R and RhlI/R on HaCaT cell line. In the case of co-culture with HaCaT cell line (Fig. 4d), *aprA* and *rhlAB* were downregulated with all the treatments, while tyrosol induced an upregulation of *toxA* and *lasB* and combination of farnesol and tyrosol upregulated *toxA*.

**Fig.5:**
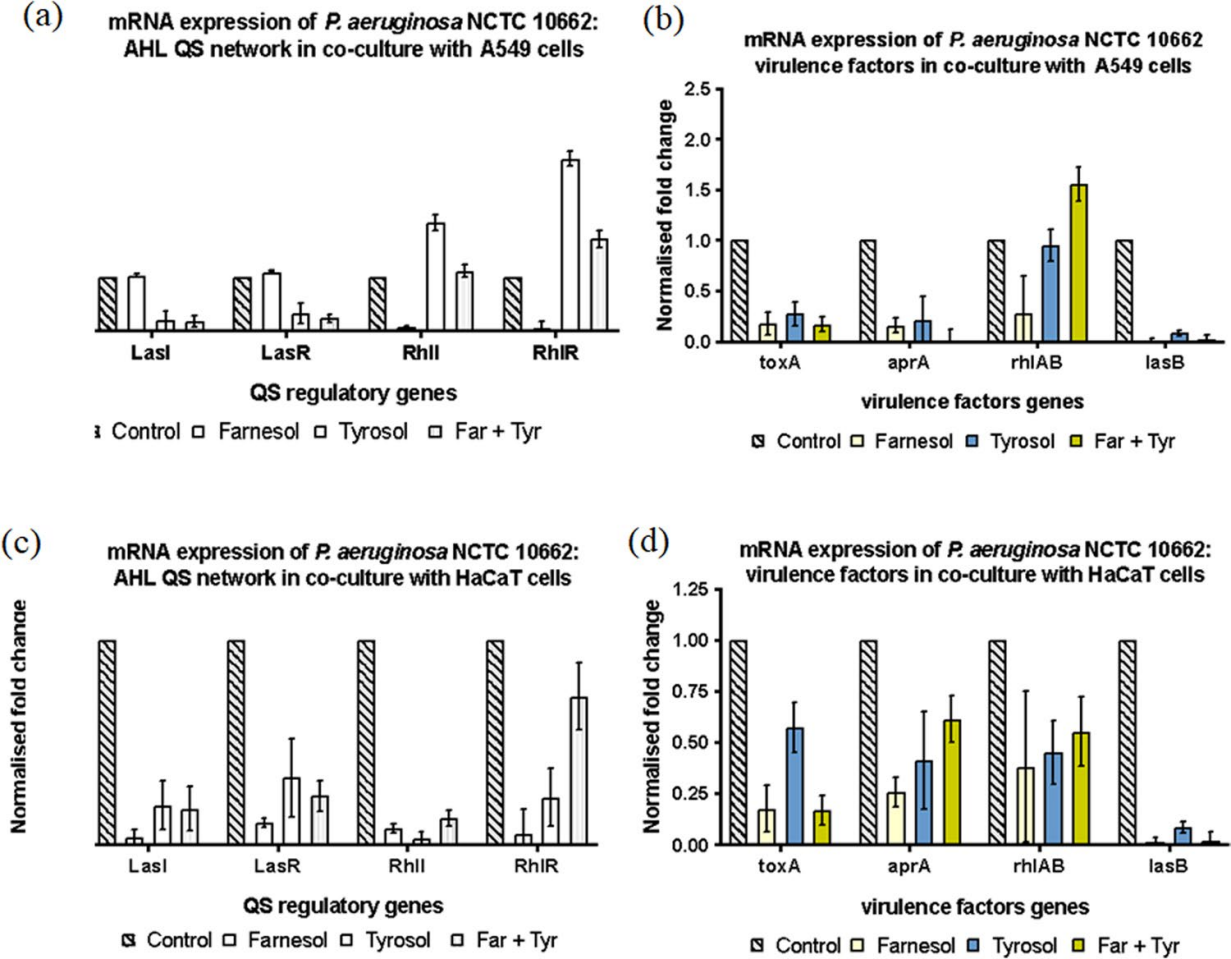
mRNA expression of AHL mediated QS circuit in *P. aeruginosa* NCTC 10,662 sessile cells grown with QQ. The fold change of mRNA for QS and virulence factors genes was determined for *P. aeruginosa* NCTC 10,662 sessile cells extracted from biofilm treated with farnesol and tyrosol after ~ 16 h growth. Results are expressed as the mean fold change (control standardised to 1.0) with error bars representing SEM (*n* = 9). Error bars represent standard deviation

Gene expression of *P. aeruginosa* NCTC 10,662 in co-culture with A549 cell line showed an upregulation of RhlI and RhlR when treated with tyrosol and combination of farnesol and tyrosol, while farnesol downregulated RhlI and RhlR (Fig. 5a). Conversely, farnesol upregulated LasI and LasR marginally, while treatment with tyrosol and combination downregulated LasI and LasR. With the exception of upregulation of *rhlAB* (combination treatment), all the other virulence factors were downregulated in co-culture with A549 (Fig. 5b). Co-culture of NCTC 10,662 on HaCaT cell line with treatment showed a consistent downregulation of LasI/R, RhlI/R and the virulence factors (Fig. 5c, d).

**Fig.6:**
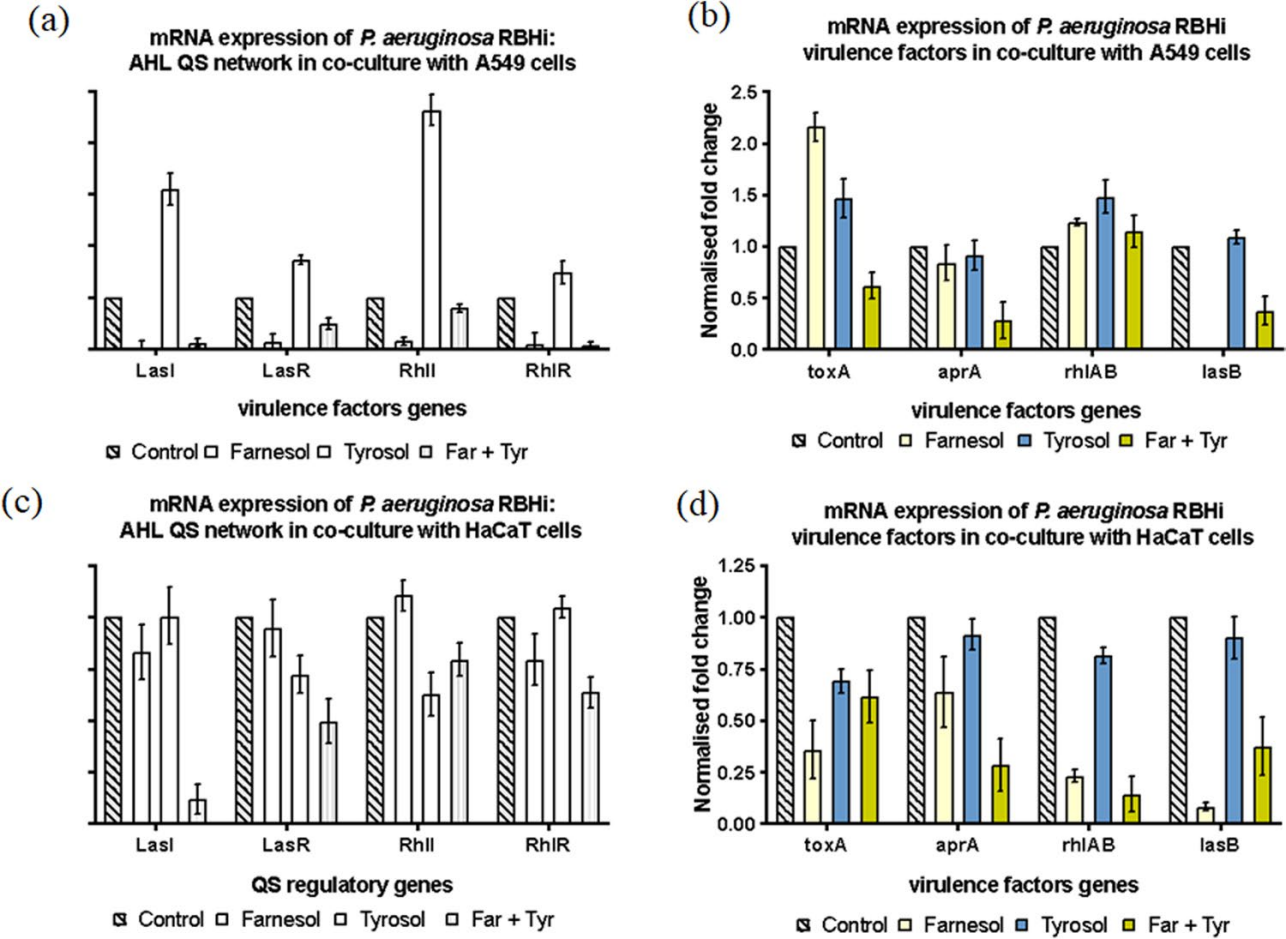
mRNA expression of AHL-mediated QS circuit in *P. aeruginosa* RBHi sessile cells grown with QQ. The fold change of mRNA for QS and virulence genes was determined for *P. aeruginosa* RBHi sessile cells (Kalgudi 2019) treated with farnesol and tyrosol after ~ 16 h growth. Results are expressed as the mean fold change (control standardised to 1.0) with error bars representing SEM (*n* = 9). Error bars represent standard deviation

Upregulation of LasI/R and RhlI/R was seen with tyrosol treatment of RBHi co-culture with A549 cell line (Fig. 6a), while the other treatments showed a pattern of downregulation. Individual treatment of farnesol and tyrosol showed an upregulation of *toxA* and *rhlAB*, while tyrosol also contributed to the upregulation of *lasB*. Combination treatment was responsible for upregulation of *rhlAB*. *aprA* remained the only virulence factor that was downregulated under combination treatment (Fig. 6b). With the exception of RhlI, which was upregulated in the co-culture of RBHi and HaCaT, all the other genes were downregulated (Fig. 6c, d).

## Discussion

At present, there is no clear study that investigates the behaviour of different phenotypes of *P. aeruginosa* in the presence of biotic surfaces, which the bacterium is known to infect. This research lays the grounds for examining the host–pathogen interactions of three *P. aeruginosa* phenotypes on two different epithelial cell lines they may infect. Additionally, each strain can have different responses to environmental stimuli, such as antagonist chemicals that inhibit biofilm formation and composition. Spot-plating experiments on bacterial adherence to A549 and HaCaT cell lines indicated that treatments containing a combination of farnesol and tyrosol significantly decreased bacterial adherence relative to the control (Fig. S3). This decreased adherence may be attributed to the secretion of exotoxins by the type III secretion system, whereby the primary function is to damage epithelial cells which leads to cytoskeletal rearrangement and results in cell lysis during the planktonic and initial colonisation stage of *P. aeruginosa* (Galle et al. 2012).

*P. aeruginosa* PAO1 adhesion to and invasion of A549 lung epithelial cells was examined by Ahmed et al. (2014) using anti-adhesion natural extracts (cranberry, dextran and soybean extracts) in combination with an antibiotic, ciprofloxacin. Their research found that when dextran and ciprofloxacin were combined, approximately 85% of the bacteria that adhered in the control experiments were unable to internalise. The combination of cranberry extract and ciprofloxacin sub-MIC inhibited the early stages of infection, i.e., adhesion and invasion, and decreased subsequent growth of bacteria. In the case of ciprofloxacin combined with soybean extract, 28% and 12% of the applied bacterial inoculum could adhere to and invade the lung epithelial cells, respectively, compared to the control.

Farnesol inhibits the synthesis of quorum sensing molecule (QSM), 2-heptyl-3-hydroxy-4-quinolone (*Pseudomonas* quinolone signal: PQS), bacterial swarming motility (McAlester et al. 2008) and PQS-regulated virulence factor pyocyanin (Cugini et al., 2007) in *P. aeruginosa*. PQS has been shown also to play a role in the development of *P. aeruginosa* biofilms. As a result, Bandara et al., (2016) hypothesised that farnesol inhibits *P. aeruginosa* biofilm growth. Based on our study, it is clear that farnesol and tyrosol display an inhibitory effect on the QS circuitry of *P. aeruginosa* as the PQS QS system is regulated by LasR and RhlR in tandem (Kostylev et al. 2019). This translates to reduced biofilm formation as depicted by reduced production of the various biopolymers that make up the biofilm (Supplemental Figs. S5 and S6). When in co-culture with the A549 and HaCaT cell lines, the formation of biofilm differed considerably along with the expression of QS and virulence genes. It is important to note that, when treated with farnesol and tyrosol, the CF isolate (RBHi) showed significantly reduced levels of gene expression in co-culture with HaCaT cells in comparison to co-culture with A549 cells. Although A549 cells are not an exact representative of CF, the CF isolate shows a highly pathogenic nature when compared to co-culture with HaCaT cells. In co-culture with the HaCaT cell lines (Supplemental Fig. S6), nearly ~ 75% reduction of biofilm was seen using combination treatment (*p* = 0.0001) against NCTC 10,662. Approximately 25% reduction was observed with RBHi (*p* = 0.0002) and ~ 45% reduction with PAO1 (*p* = 0.0027). However, farnesol-inhibited biofilm formation was not significant (*p* = 0.325). On the other hand, inhibition of biofilm formation under tyrosol and combination of the two QQ agents were significant (*p* = 0.023 and 0.0002, respectively) (Supplemental Fig. S6). The eDNA and protein content of the biofilm produced by the mucoid strain on the HaCaT cell line against all three treatments remained identical to the control, with the exception of NCTC 10,662, which appeared to be affected by the combination treatment, showing a 40% reduction (*p* = 0.0028) in eDNA content and a 30% reduction (*p* = 0.001) in protein content of the biofilm. Furthermore, the reduction in eDNA and protein content was not found to be significant when compared between farnesol and tyrosol. When RBHi was treated with a combination of farnesol and tyrosol, reduction in carbohydrate content (55%) was observed. With the combined treatment, RBHi was also the only strain to exhibit an 18% decrease in alginate content (*p* = 0.034). Comparing the two cell lines (Supplemental Figs. S5 and S6), all the phenotypes of *P. aeruginosa* demonstrated a stronger affinity towards A549 cell line compared to HaCaT cell line, as shown by its ability to form biofilms and secrete EPS. Overall, farnesol and tyrosol combined treatment was more successful against *P. aeruginosa* biofilm. However, tyrosol was the most successful of the individual treatments.

Figure 3 a and b reveal that a higher rate of pro-inflammatory effect is achieved when bacterial cells are in contact with HaCaT cells when compared with A549. This also indicates the sensitivity of HaCaT cells towards inflammatory agents, compared to A549 cells. The production of IL-8 by the epithelial cells is an important indicator of host response and production of IL-8 is generally associated with acute and chronic infections. There is little data comparing IL-8 secretion by two different cell lines when exposed to biofilm formed by *P. aeruginosa*; comparative data on IL-8 secretion as an inflammatory response in co-culture with three distinct strains of biofilm forming *P. aeruginosa* is lacking. In the case of NCTC 10,662, the reduction in IL-8 output after treatment with farnesol was found to be significant, while the others were not. This may be due to the static nature of the co-culture, as well as the fact that farnesol and tyrosol are both QS molecules found in fungi. As a result, they can cause a similar reaction and increase IL-8 secretion by epithelial cells. Low amounts, on the other hand, did not result in an increase in IL-8 output. The static growth model of this assay limits its ability to explain the antagonistic effect of farnesol and tyrosol in combination treatment. To test the effect of farnesol and tyrosol on *P. aeruginosa* biofilm inhibition, a flow model, similar to a microfluidic chip, or a robust co-culture model using 3D tissue culture of mammalian cells should be used.

Both adhesion and formation of biofilms depend primarily on motility (Sun et al., 2017). Swimming, swarming and twitching are three forms of motility in *P. aeruginosa* that are positively controlled by the LasIR and RhIR QS systems; las regulating swimming, swarming and twitching; and rhI controlling swarming and twitching (Yeung et al., 2009). Abdel-Rhman et al., (2020) found that tyrosol (1/4 ×—1/16 × MIC) significantly decreased both swimming and swarming motility, but had no effect on twitching motility. Our study shows that regulation of gene expression differs significantly between strains and in the presence of similar treatments (QQ) (Figs. 5 and 6). This is evident in varying levels of gene expression seen for LasI/R and Rhll/R. Expression of virulence progressively increases with increase in mucoidity of *P. aeruginosa*. This is in parallel with developed *P. aeruginosa* biofilms and chronic infections.

The study of gene expression levels of bacterial communication and virulence production in a host–pathogen model has contributed to the current understanding of the creation of potential novel therapies. Future modifications to this model can better reflect in vivo infection conditions, allowing researchers to better define host–pathogen interactions and develop new therapeutic regimens to combat biofilm formation.

## Supplementary Information

Below is the link to the electronic supplementary material.

Supplementary file1 (PDF 820 KB)

## Data Availability

The authors confirm that all relevant data are included in this article and its supplementary information files.
