# Supplementary material for: Quorum quenchers affect the virulence regulation of non-mucoid, mucoid and heavily mucoid biofilms co-cultured on cell lines
Source: Appl Microbiol Biotechnol. 2021 Oct 30;105(23):8853–68. doi: 10.1007/s00253-021-11638-8 (PMC8590680; doi:10.1007/s00253-021-11638-8)
Supplement: Supplementary file 1 — Supplementary file1 (PDF 820 KB) [file 253_2021_11638_MOESM1_ESM.pdf]

# SUPPLEMENTARY FIGURES

Journal: **Applied Microbiology and Biotechnology**

Article Title: **Quorum quenchers affect the virulence regulation of non-mucoid, mucoid and heavily mucoid biofilms co-cultured on cell lines**

Authors: Rachith Kalgudi[a, *], Roya Tamimi[a], Godfrey Kyazze[a], Tajalli Keshavarz[a]

[a]School of Life sciences, University of Westminster, 115 New Cavendish Street, London, W1W 6UW, United Kingdom

* Corresponding author**:** E-mail: rachith.kalgudi@gmail.com

(a)

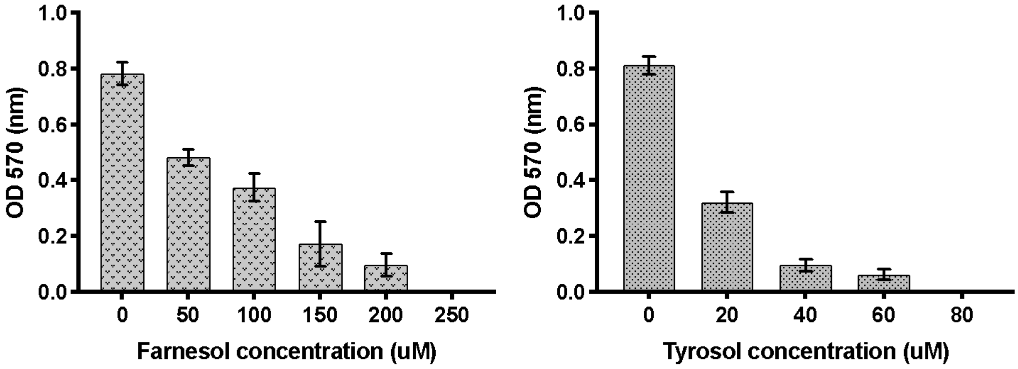


(b)

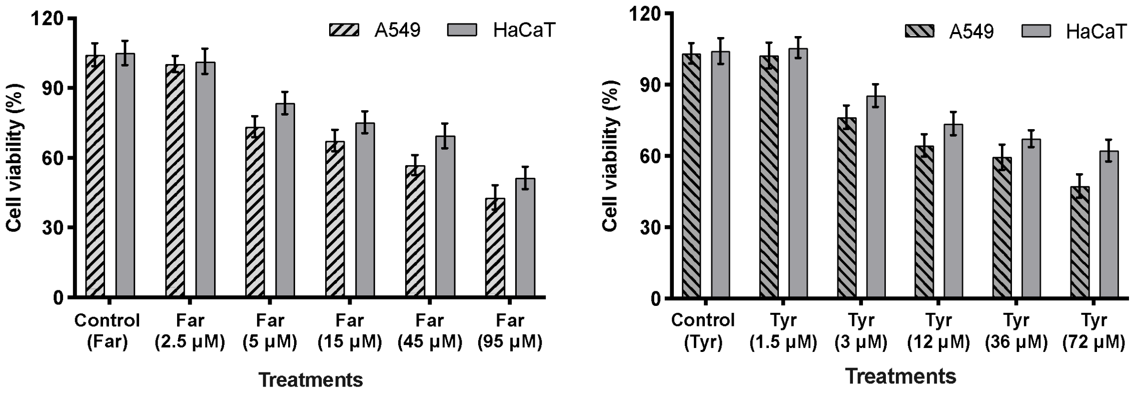


(c)

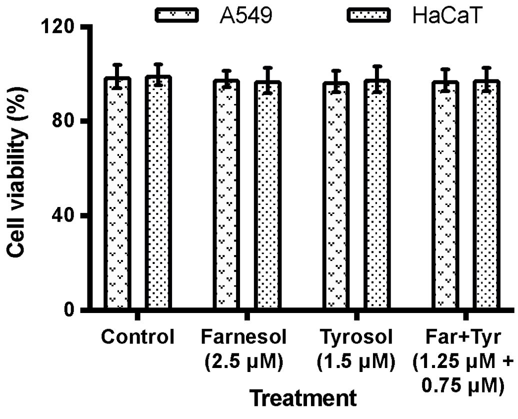


**Fig.S1** (a) $MIC_{50}$ of farnesol and tyrosol against *P. aeruginosa* PAO1. (b) Range of farnesol and tyrosol doses used against A549 and HaCaT cell lines. (c) Sub-$MIC_{50}$ doses of tyrosol and farnesol, individually and in combination against A549 and HaCaT cells. PBS was used as control (n=5). Error bars represent standard deviation.

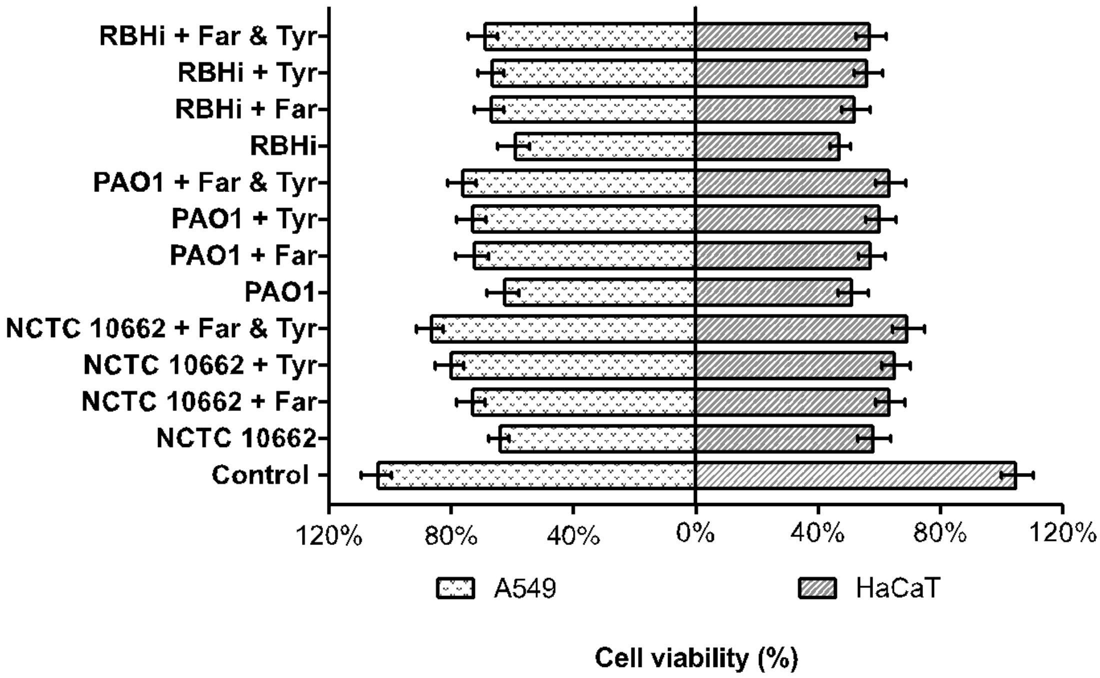


**Fig.S2** Viability of A549 (left) and HaCaT (right) cells in co-culture with bacteria, with treatment using farnesol and tyrosol as QQ and untreated cells based on the MTT assay (n=3). Error bars represent standard deviation

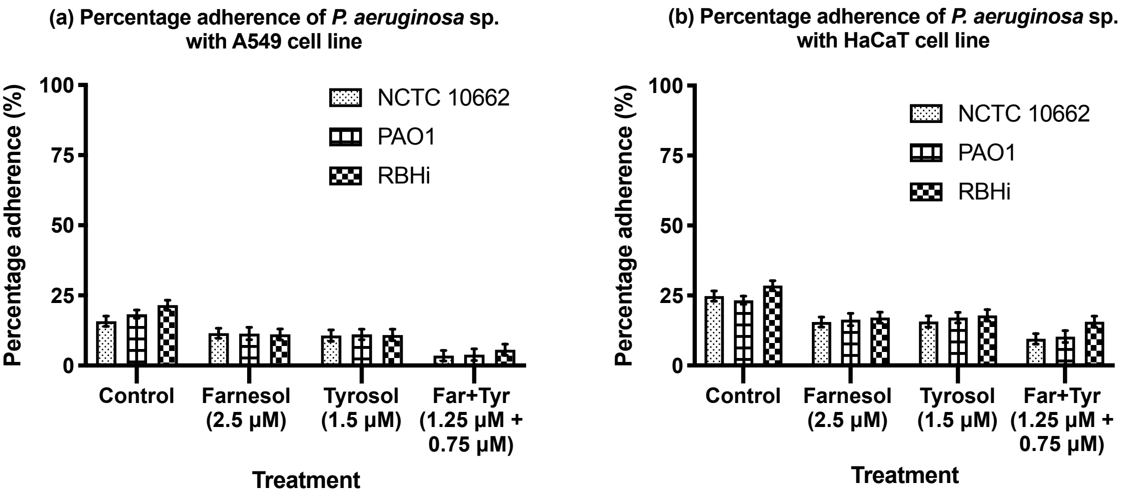


**Fig.S3** Adherence of *P. aeruginosa* sp. cells onto the surface of (a) A549 and (b) HaCaT cell lines. The percentage is based on calculating the adhered CFU vs the initial cell count in the bacterial inoculum. All the treatments, individual and in combination reduced the percentage adherence of bacterial cells to the mammalian cells ($p$= 0.0001 and 0.0001) (n=5). Error bars represent standard deviation

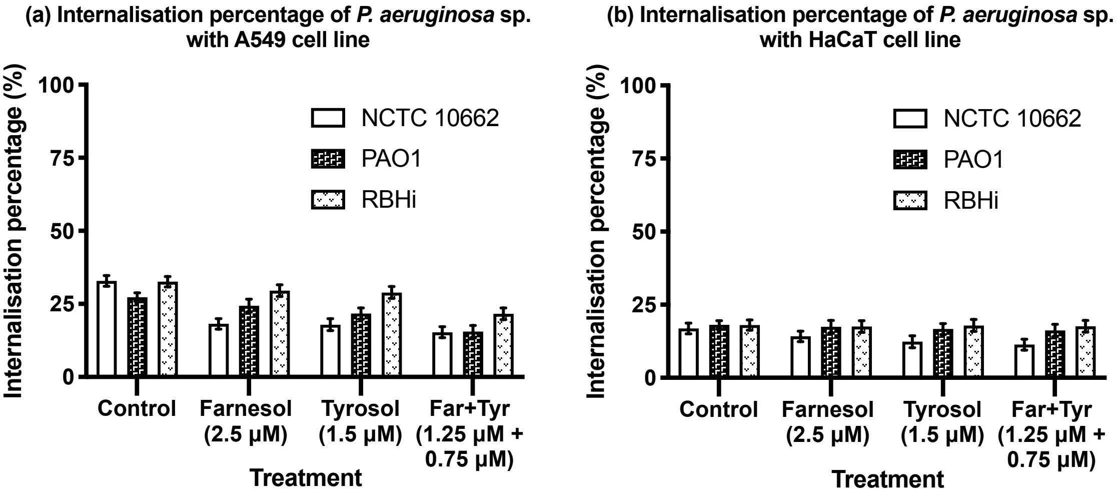


**Fig.S4** Internalisation of *P. aeruginosa* sp. cells into cells of (a) A549 and (b) HaCaT. The percentage is based on calculating the internalised CFU vs the initial cell count in the bacterial inoculum (n=5). Error bars represent standard deviation

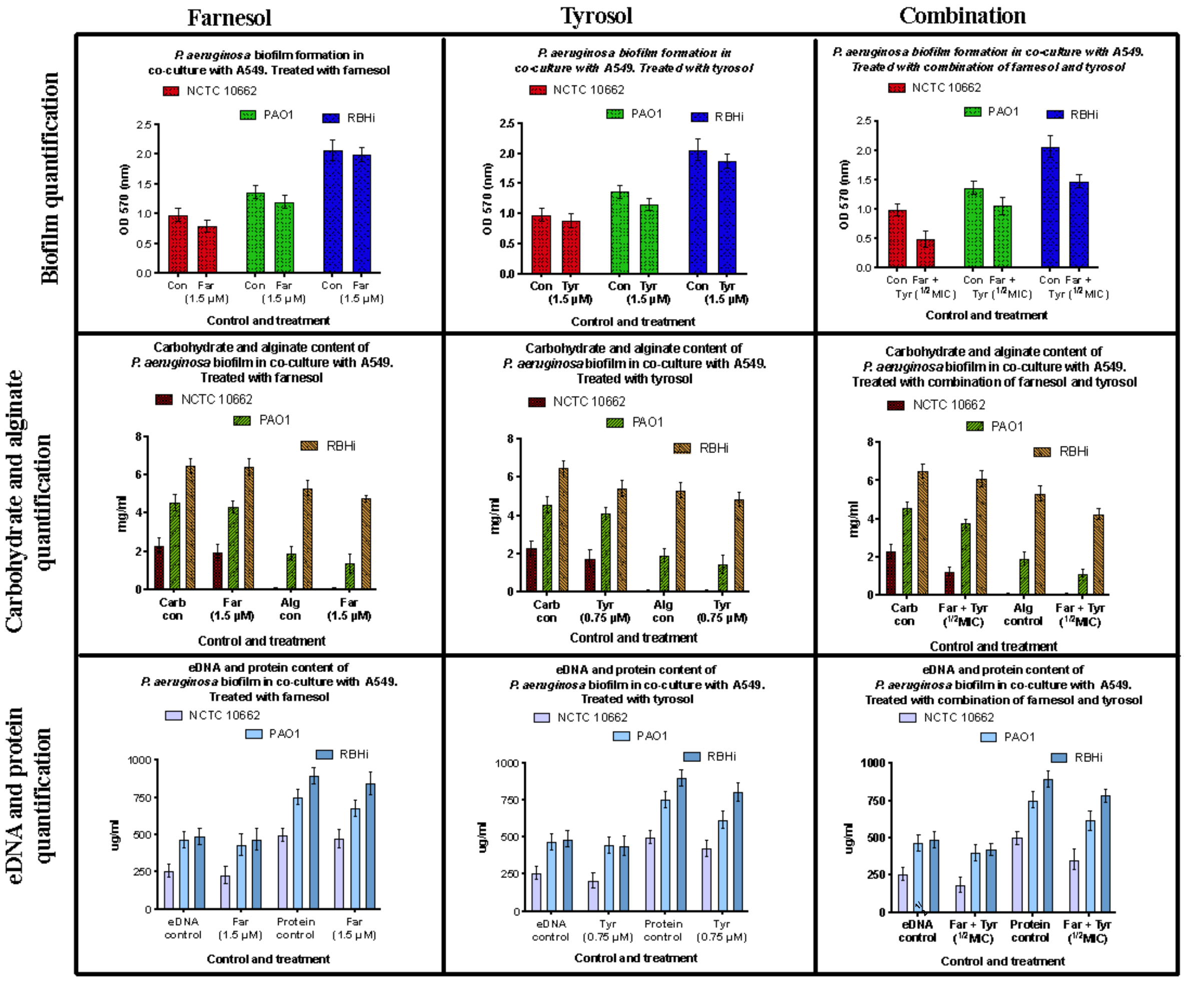


**Fig.S5** Synergistic and individual effects of farnesol and tyrosol on *P. aeruginosa* sp biofilm formation, architecture and virulence factor production when grown in co-culture with A549 cell line (n=5). Error bars represent standard deviation

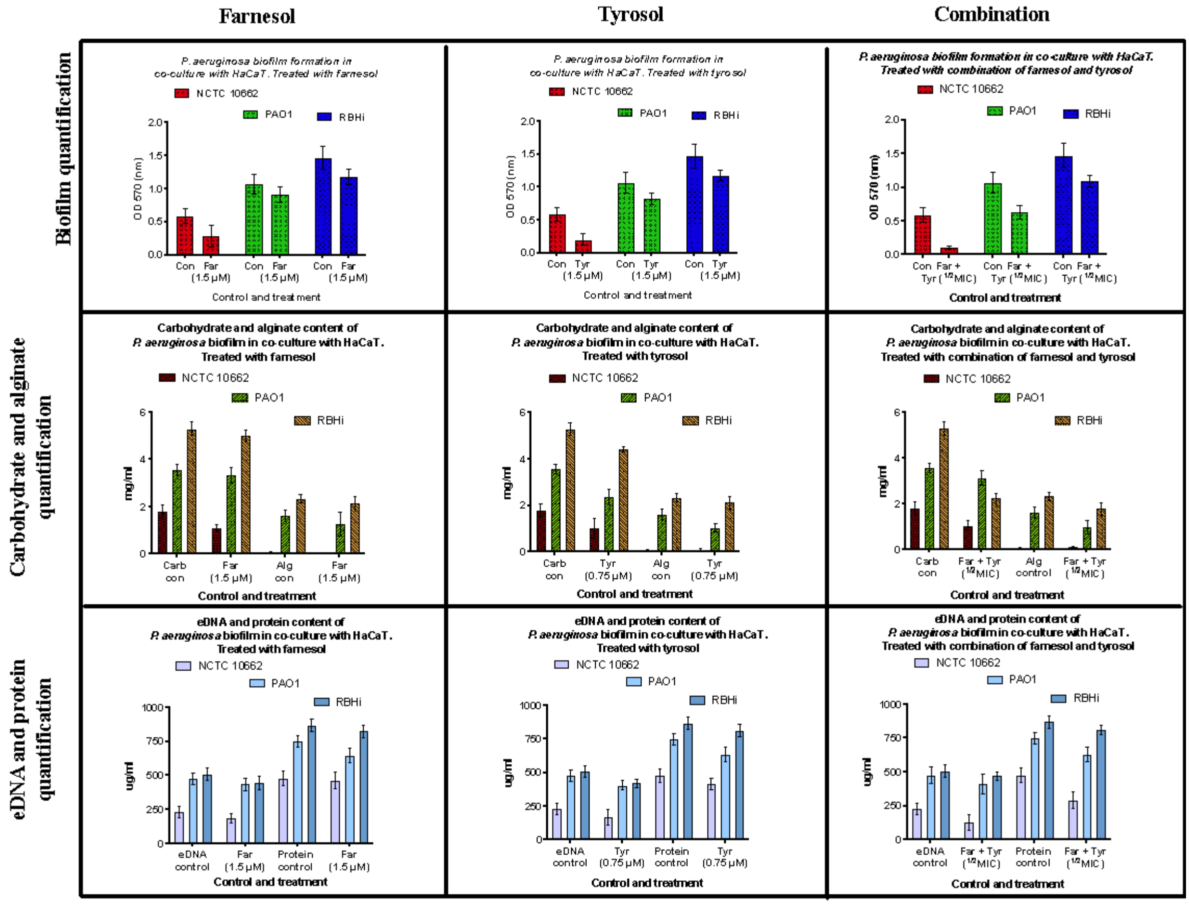


**Fig.S6** Synergistic and individual effects of farnesol and tyrosol on *P. aeruginosa* sp biofilm formation, architecture and virulence factor production when grown in co-culture with HaCaT cell line (n=5). Error bars represent standard deviation
